# Temperature dependent control of the solubility of gallium nitride in supercritical ammonia using mixed mineralizer

**DOI:** 10.1186/s13065-018-0501-7

**Published:** 2018-12-03

**Authors:** Daisuke Tomida, Kiyoshi Kuroda, Kentaro Nakamura, Kun Qiao, Chiaki Yokoyama

**Affiliations:** Institute of Multidisciplinary Research for Advanced Materials, Tohoku University, 2-1-1 Katahira, Aoba-ku, Sendai, 980-8577 Japan

**Keywords:** Ammonothermal, Solubility, Gallium nitride, Acidic mineralizer, Supercritical ammonia

## Abstract

Using a mass-loss method, we investigated the solubility change of gallium nitride (GaN) in supercritical ammonia with mixed mineralizers [ammonium chloride (NH_4_Cl) + ammonium bromide (NH_4_Br) and NH_4_Cl + ammonium iodide (NH_4_I)]. The solubilities were measured over the temperature range 450–550 °C, at 100 MPa. The solubility increased with NH_4_Cl mole fraction at 450 °C and 100 MPa. The temperature dependence of the solubility curve was then measured at an equal mole ratio of the two mineralizers. The slope of the solubility–temperature relationship in the mixed mineralizer was between those of the individual mineralizers. These results show that the temperature dependence of the solubility of GaN can be controlled by the mineralizer mixture ratio. The results of the van’t Hoff plot suggest that the solubility species were unchanged over the investigated temperature range. Our approach might pave the way to realizing large, high-quality GaN crystals for future gallium-nitride electronic devices, which are increasingly on demand in the information-based age.

## Introduction

In an increasingly information-based society, high-speed wireless communications systems with massive information-transmission capability are expected as a ubiquitous network technology in the near future. However, to realize such systems, the power and operating frequency of electronic devices need to be increased. Gallium-nitride devices offer a promising solution, as their power and frequency is expected to exceed those of Si-based devices. However, these devices require a large-diameter, high-quality GaN bulk single-crystal substrate, which does not yet exist. Although heteroepitaxial growth can be carried out on sapphire substrate by the hydride vapor phase epitaxy (HVPE) method, the lattice mismatch increases the dislocation density of the growth. For this reason, there has been a race to develop bulk GaN single-crystal substrates using various methods. Single-crystal GaN is mainly grown by the Na flux method [1, 2] or the ammonothermal method [3–11]. The ammonothermal method is promising for its relatively mild crystal growth conditions and the ease of up-scaling the equipment.

Previously, we reported a GaN crystal growth rate exceeding the minimum requirements of industrial application (100 μm/day) using the ammonothermal method with NH_4_I as the mineralizer [7]. However, because the GaN solubility rapidly increases around 530 °C, the supersaturation level was difficult to control by this approach [12]. Although supersaturation is a driving force for crystal growth, spontaneous nucleation overcomes crystal growth under excessive supersaturation conditions. In fact, when NH_4_I is used as the mineralizer, a large number of needle crystals are deposited on the inner wall of the autoclave [13]. Changing the temperature difference between the raw material dissolution region and the crystal growth region, the type of mineralizer, and other factors can control the supersaturation level. Controlling the temperature dependence of GaN solubility by altering the mineralizer-mixing ratio would be very useful for ammonothermal crystal growth, because mineralizer addition is an easily adjustable parameter.

Several researchers have measured the solubility of GaN in supercritical ammonia with a single mineralizer [12, 14–19]. However, the solubility of GaN in supercritical ammonia with mixed mineralizers has not been reported yet. Thus, the present study investigates the change in the solubility of GaN in supercritical ammonia under addition of a mixed mineralizer, and whether the mixing ratio can control the temperature dependence of the solubility.

## Materials and methods

GaN crystals were grown by HVPE. The mineralizers NH_4_Cl (purity 99.5%), NH_4_Br (purity 99.0%), and NH_4_I (purity 99.5%) were purchased from Wako Pure Chemical Industries (Japan), and dried at 100 °C for 24 h before use. Ammonia (NH_3_, purity 99.999%) was obtained from Japan Fine Products Co. Ltd (Japan).

The solubility was measured by the mass-loss method, as described in our previous paper [18]. The uncertainties in the temperature and pressure values were ± 2 °C and ± 2 MPa, respectively. The composition of the sample mixtures was determined by weighing the chemicals at the desired molar ratio. The estimated measurement uncertainty in the solubility was within ± 10%.

## Results

**Fig. 1:**
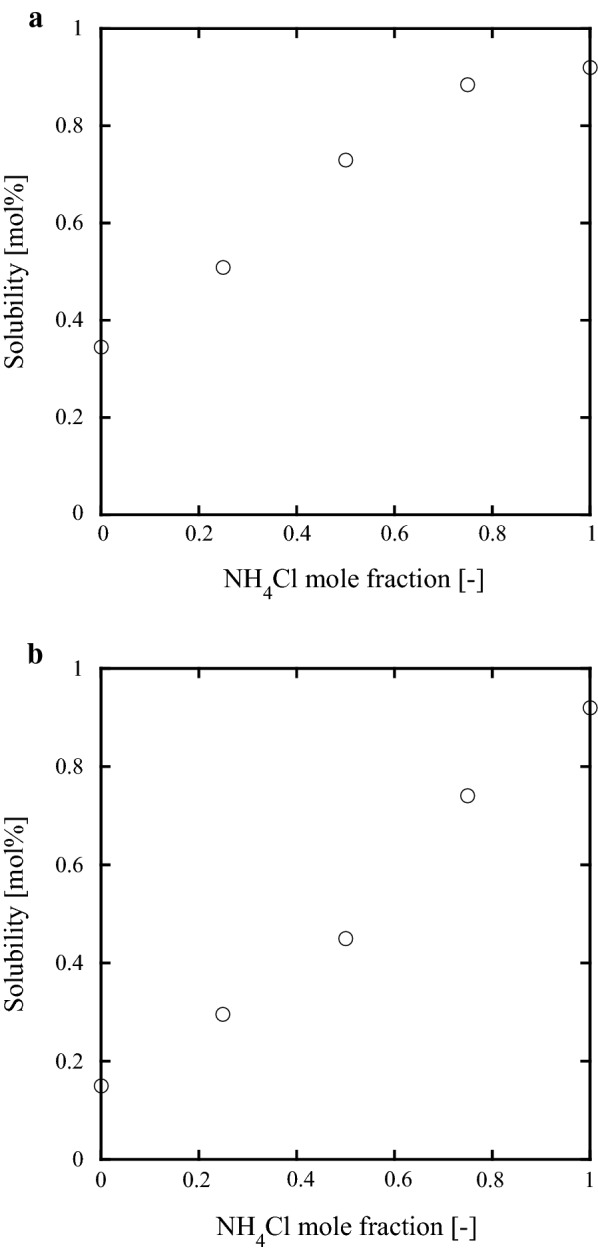
Mineralizer-composition dependence of GaN solubility in supercritical ammonia (450 °C, 100 MPa, and 3.1 mol% mixed mineralizer): **a** NH_4_Cl + NH_4_Br; **b** NH_4_Cl + NH_4_I

The measured solubilities of GaN in supercritical ammonia with mixed mineralizer compositions of NH_4_Cl + NH_4_Br and NH_4_Cl + NH_4_I are given in Tables 1 and 2. Panels (a) and (b) of Fig. 1 show the mineralizer-composition dependence of the GaN solubility in supercritical ammonia in the presence of NH_4_Cl + NH_4_Br and NH_4_Cl + NH_4_I, respectively. In both systems, the temperature and pressure were 450 °C and 100 MPa respectively, and the mixed-mineralizer concentration was 3.1 mol%. In the NH_4_Cl + NH_4_Br mixture, the GaN solubility curve became gradually convex with increasing molar fraction of NH_4_Cl, but in the NH_4_Cl + NH_4_I mineralizer, it was an almost-linear function of NH_4_Cl molar fraction.

**Table 1:** Solubility of GaN in supercritical ammonia with NH_4_Cl + NH_4_Br mixed mineralizer

Temperature (°C)	Pressure (MPa)	Concentration of mineralizer (mol%)	Mole fraction of NH_4_Cl (−)	Solubility (mol%)
450	100	3.1	0.00	0.35
450	96	3.1	0.25	0.51
450	101	3.1	0.50	0.73
450	101	3.1	0.75	0.88
450	100	3.1	1.00	0.92
415	102	3.0	0.50	0.46
440	101	3.1	0.50	0.66
460	102	3.1	0.50	0.79
500	99	2.9	0.50	1.05
550	103	3.0	0.50	1.23

**Table 2:** Solubility of GaN in supercritical ammonia with NH_4_Cl + NH_4_I mixed mineralizer

Temperature (°C)	Pressure (MPa)	Concentration of mineralizer (mol%)	Mole fraction of NH_4_Cl (−)	Solubility (mol%)
450	100	2.9	0.00	0.15
450	98	3.1	0.25	0.30
450	100	3.1	0.50	0.45
450	96	3.2	0.75	0.74
450	100	3.1	1.00	0.92
495	101	3.1	0.50	0.58
515	101	3.1	0.50	0.73
550	102	3.1	0.50	1.20

**Fig. 2:**
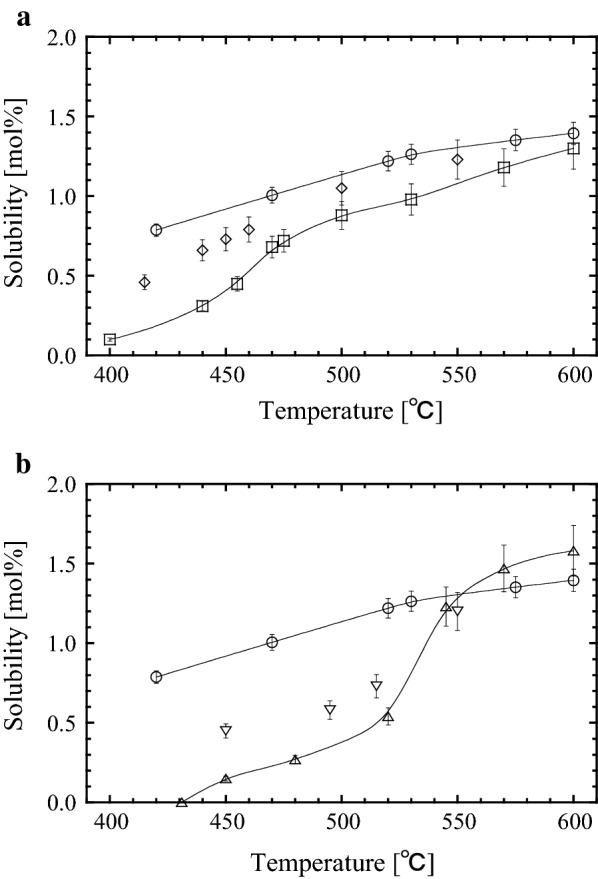
Temperature dependence of GaN solubility in supercritical ammonia with different mineralizers (100 MPa, 3.1 ± 0.1% mineralizer): NH_4_Cl, circle (from previous work [18]); NH_4_Br, square (from previous work [12]); NH_4_I, triangle (from previous work [12]); NH_4_Cl + NH_4_Br (equal mole ratio), rhombus (present study); NH_4_Cl + NH_4_I (equal mole ratio), nabla (present study): **a** NH_4_Cl, NH_4_Br, NH_4_Cl + NH_4_Br; **b** NH_4_Cl, NH_4_I, NH_4_Cl + NH_4_I

Next, we investigated the temperature dependence of the solubility curve in a 1:1 molar ratio mixture. The results for the NH_4_Cl + NH_4_Br and NH_4_Cl + NH_4_I systems are shown in panels (a) and (b) of Fig. 2, respectively. The curve for the NH_4_Cl + NH_4_Br system lies between those of the single NH_4_Cl and NH_4_Br mineralizers. Similarly, the curve of the NH_4_Cl + NH_4_I system almost lies between those of the single NH_4_Cl and NH_4_I mineralizers.

## Discussion

According to our results, the slope of the GaN solubility curve can be changed by adding a mixed mineralizer, and can be controlled by the mixing ratio of the mineralizers.

In our previous studies [12, 18], the solubility of GaN in supercritical ammonia with acidic mineralizers (NH_4_Cl, NH_4_Br, and NH_4_I) was described by the van’t Hoff equation. Here we apply this equation to the solubility of GaN in supercritical ammonia with mixed mineralizers (NH_4_Cl + NH_4_Br, NH_4_Cl + NH_4_I).

In general, the van’t Hoff equation extracts the heat of solution from the temperature dependence of the solubility. The equation is given by

1 $${ \ln }s = - \Delta H/RT + C,$$

where *s* is the solubility in mol%, ∆*H* is the heat of solution in kJ/mol, *R* is the gas constant in J/(mol K), *T* is the temperature in K, and *C* is a constant. The compositions of the solvent and the dissolving species are assumed fixed under all experimental conditions.

**Fig. 3:**
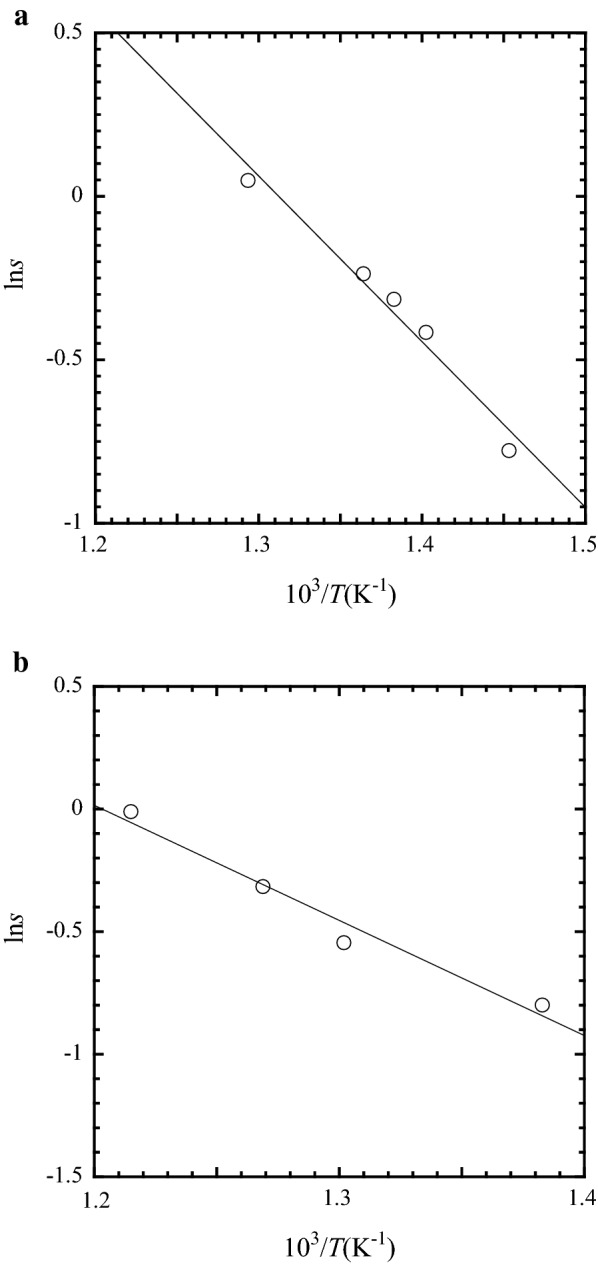
Relationship between ln*s* and 10^3^/*T* for the solubility of GaN in supercritical ammonia with different mineralizer mixtures (100 MPa, and 3.1 mol%): **a** NH_4_Cl + NH_4_Br (equal mole ratio); **b** NH_4_Cl + NH_4_I (equal mole ratio)

Figure 3 plots the logarithmic solubility of GaN in the NH_4_Cl + NH_4_Br and NH_4_Cl + NH_4_I systems against the reciprocal of the absolute temperature.

The slope of the plot is almost constant in both systems, suggesting that the solubility species were unchanged over the investigated temperature range.

From the slopes of the straight lines in Fig. 3a and b, the heats of solution of GaN in supercritical ammonia were respectively calculated as follows.

- ∆*H* = 42.1 kJ/mol for NH_4_Cl + NH_4_Br
- ∆*H* = 39.0 kJ/mol for NH_4_Cl + NH_4_I.

**Fig. 4:**
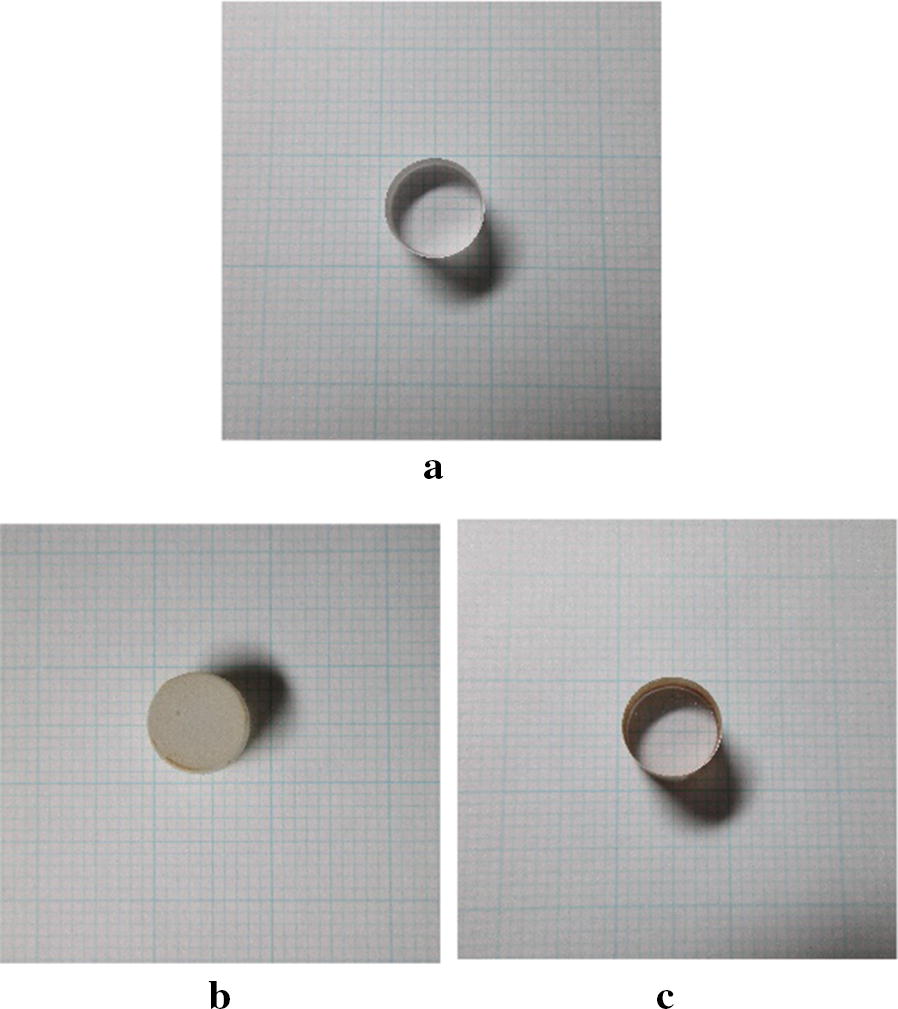
Photographs showing the appearance of sapphire glass **a** before corrosion resistance test **b** after corrosion resistance test using NH_4_F mineralizer (**b**) after corrosion resistance test using NH_4_Cl mineralizer: corrosion resistance test conditions were 450 °C, 102 MPa, 2.0 ± 0.1 mol% mineralizer concentration

Compared with data from Schimmel et al. [19], which were measured in situ, our solubility values [18] are high. Therefore, we examined the differences between our measurements and theirs. To improve X-ray transmission, Schimmel et al. used sapphire glass, which they state exhibits corrosion resistance under acidic ammonothermal conditions using NH_4_F or NH_4_Cl as a mineralizer. To investigate this, we performed a corrosion resistance test with sapphire glass under very similar conditions to those used by Schimmel et al. in their solubility experiments. These conditions were a temperature of 450 °C, pressure of 102 MPa, mineralizer concentration of 2.0 mol%, and reaction time of 6 h. We photographed the sapphire glass before and after the corrosion resistance test (Fig. 4). When NH_4_F was used as a mineralizer, the sapphire glass corroded and lost its transparency. By contrast, when NH_4_Cl was used as a mineralizer, the sapphire glass transparency was maintained. We also weighed the sapphire glass before and after the experiments (Table 3). With NH_4_F, the mass of the sapphire glass decreased, which indicated it corroded. With NH_4_Cl, although the transparency was maintained, the mass of the sapphire glass decreased slightly, which indicated that it also corroded a small amount. Sapphire glass clearly corroded in the 6-h NH_4_F reaction. Therefore, the solubility values from Schimmel et al. could be lower than the actual values because the mineralizer concentration decreased. However, when NH_4_Cl was used as a mineralizer, although the sapphire glass corroded slightly, it did not corrode enough to affect the solubility data. In this case, the differences between the two sets of solubility data cannot be explained by the use of sapphire glass.

**Table 3:** Mass of sapphire glass before and after the corrosion resistance test (conditions: 450 °C, 102 MPa, mineralizer concentration 2.0 mol%, and 6 h)

Mineralizer	Mass of sapphire glass before experiment (g)	Mass of sapphire glass after experiment (g)
NH_4_F	1.1998	1.1814
NH_4_Cl	1.1930	1.1894

Pimputkar et al. [20] investigated the possibility of Ga sinking into Mo as a contributor to the decreased feed rate of raw material in experiments using Mo capsules. Therefore, we examined the possibility that our solubility data were high because of Ga sinking into Pt. First, we placed polycrystalline GaN in a Pt crucible and heated it in a nitrogen atmosphere at 400–600 °C for 100 h. We measured the masses of the Pt crucible and polycrystalline GaN before and after the experiment (Table 4). There were no changes in the masses of Pt crucible and polycrystalline GaN at any temperature, and no indication that Ga sinking into Pt occurred. Next, we placed a Pt plate on the bottom of the autoclave and polycrystalline GaN on the plate, and attempted to measure the solubility. The experimental conditions were a temperature of 420 °C, pressure of 101 MPa, mineralizer concentration of 3.0 mol%, and autoclave heating time of 100 h. The solubility (0.76 mol%) agreed with the previous measurement (0.79 mol%) [18] within the measurement uncertainty. We did not observe any mass change in the Pt plate after the experiment (Table 5), and it does not seem possible that our solubility data were high because Ga sank into Pt. When Pimputkar et al. considered the possibility of Ga sinking into Mo, they found that Mo and Ga did not form an alloy. As in the case of using Mo capsules, Ga did not sink into Pt and it did not affect the solubility data.

**Table 4:** Mass of platinum (Pt) crucible and polycrystalline gallium nitride (GaN) before and after heating for 100 h under a nitrogen atmosphere

Temperature (°C)	Mass of Pt crucible before experiment (g)	Mass of Pt crucible after experiment (g)	Mass of polycrystalline GaN before experiment (g)	Mass of polycrystalline GaN after experiment (g)
400	4.0347	4.0347	0.3341	0.3341
500	4.0347	4.0347	0.3341	0.3341
600	4.0347	4.0347	0.3341	0.3341

**Table 5:** Mass of platinum (Pt) plate before and after solubility measurements (conditions: 420 °C, 101 MPa, mineralizer concentration 3.0 mol%)

Mineralizer	Mass of Pt plate before experiment (g)	Mass of Pt plate after experiment (g)
NH_4_Cl	0.0810	0.0810

In their experimental procedure, Schimmel et al. released ammonia to adjust the pressure if necessary. However, because NH_4_Cl easily dissolves in ammonia, NH_4_Cl would also be released with the ammonia. Therefore, the mineralizer amount-of-substance fraction could not be accurate. They also did not weigh the ammonia, and there is uncertainty as to the amount of ammonia they used. In the experimental section, they describe that ammonia introduced into the autoclave up to fill factor of 60%. But, they do not mention the uncertainty around the amount of ammonia. In summary, it is not clear why our solubility data differ from those of Schimmel et al.

## Conclusions

We investigated the change in solubility of gallium nitride (GaN) in supercritical ammonia in the presence of mixed mineralizers. The solubility curve of the NH_4_Cl + NH_4_Br system gradually became convex with increasing NH_4_Cl molar fraction. In contrast, the GaN solubility in the NH_4_Cl + NH_4_I system increased almost linearly with NH_4_Cl molar fraction. The temperature dependence of the solubility was investigated in 1:1 molar ratio mixtures. The slope of the dependence in the NH_4_Cl + NH_4_Br (NH_4_Cl + NH_4_I) system was intermediate between the slopes of the systems with single NH_4_Cl mineralizer and single NH_4_Br (NH_4_I) mineralizer. These results show that adding a mixed mineralizer to the system changes the slope of the solubility curve. Moreover, the GaN solubility can be controlled by the mixing ratio of the individual mineralizers.
